# Going ‘meta’: a systematic review of metacognition and functional neurological disorder

**DOI:** 10.1093/braincomms/fcaf014

**Published:** 2025-01-13

**Authors:** Anna Sadnicka, Ann-Marie Strudwick, John P Grogan, Sanjay Manohar, Glenn Nielsen

**Affiliations:** Neurosciences and Cell Biology Research Institute, St George’s University of London, London SW17 0RE, UK; Gatsby Computational Neuroscience Unit, University College London, London W1T 4JG, UK; Department of Clinical and Movement Neurosciences, University College London, London WC1N 3BG, UK; Neurosciences and Cell Biology Research Institute, St George’s University of London, London SW17 0RE, UK; Trinity Institute of Neuroscience, Trinity College Dublin, Dublin D02 PX31, Ireland; Department of Experimental Psychology, University of Oxford, Oxford OX2 6GG, UK; Neurosciences and Cell Biology Research Institute, St George’s University of London, London SW17 0RE, UK

**Keywords:** metacognition, review, functional neurological disorders, meta

## Abstract

In functional neurological disorder (FND), there is a fundamental disconnect between an apparently intact nervous system and the individuals’ ability to consistently perform motor actions, perceive sensory signals and/or access effective cognition. Metacognition, the capacity to self-evaluate cognitive performance, appears highly relevant to FND pathophysiology. Poor metacognition is a potential mechanism via which abnormal models of self and the state of the world could arise and persist unchecked. There is therefore a justified enthusiasm that studies of metacognition may give substance to FND’s intangible nature. However, many assume an impairment in metacognition even though experimental studies are still in their infancy. This systematic review provides an analytical checkpoint of the evidence after the first five years of experimental work. We firstly summarize current methods for testing metacognition, prerequisite knowledge that allows readers to independently evaluate the evidence. Using the Preferred Reporting Items for Systematic Reviews and Meta-Analyses (PRISMA) guidelines, we then screened the 21 articles on this topic and review the experimental data of the eight studies that specifically tested metacognition in subjects with FND. Questionnaire metrics used to estimate global metacognition and general confidence in FND revealed a mixed picture of low or normal confidence. Of the five studies that used performance-controlled metrics, the gold-standard to estimate local metacognition in FND, four found metacognition to be equivalent to healthy controls and one paper supported impaired metacognition. We consequently try and broaden the debate and discuss alternative headline scenarios. We review how positive studies may offer insight and debate whether null studies could represent false negatives. However, since most studies find equivalent metacognition to controls, we also discuss whether metacognition could be intact and how this could inform mechanistic models of FND and have potential clinical utility. In summary, this review highlights signal of interest within the data, exposes current limitations and flags the many open questions.

## Teaser

Sadnicka *et al.* overview experimental data that has examined metacognition in patients with functional neurological disorders (FNDs). Interestingly, most studies that independently model metacognition reveal intact metacognition. Whether there is a signature metacognitive profile in FND is therefore highly uncertain, and alternative scenarios and their potential clinical relevance are debated.

## Graphical Abstract

**Graphical Abstract:**
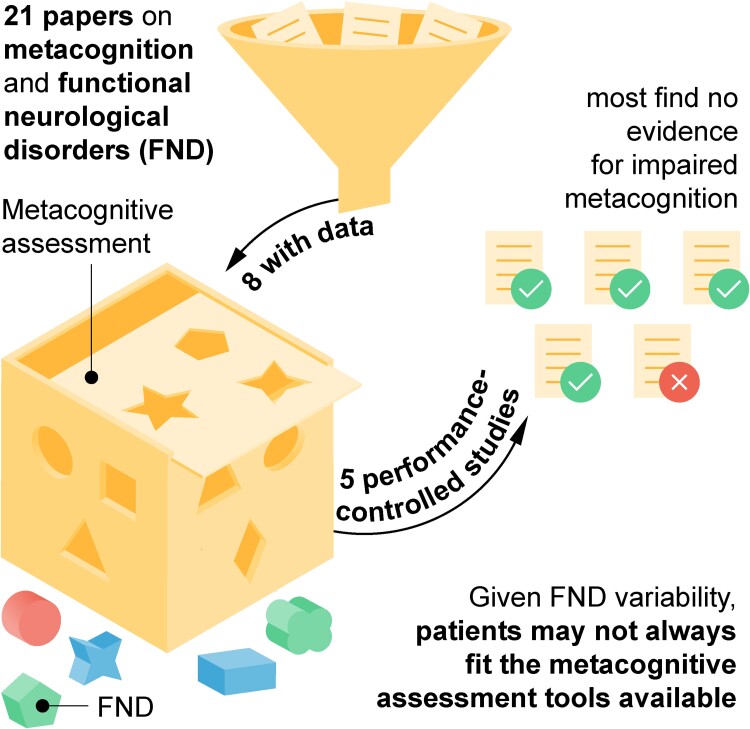

## Introduction

Functional neurological disorder (FND) is one of the most common causes of disabling cognitive, sensory and motor symptoms in neurology.^1^ Best understood within nuanced biopsychosocial models, a complex set of predisposing factors interact with physical or psychological events to precipitate and perpetuate symptoms.^1-3^ Symptoms of FND are varied yet often cluster in patients.^2,4^ There is increasing recognition that there are shared mechanisms at the root of FND, mechanistic features that are common to diverse symptomatology across functional motor, sensory, cognitive and seizure disorders.^4,5^ At the heart of perspectives on FND are disrupted attention, self-agency and emotion/threat processing.^6^ Hierarchical neural processing appears to be perturbed with abnormally precise top-down priors dominating lower-level processing disturbing our usual perceptual inference of the state of our brain, body and world.^7,8^

**Figure 1:**
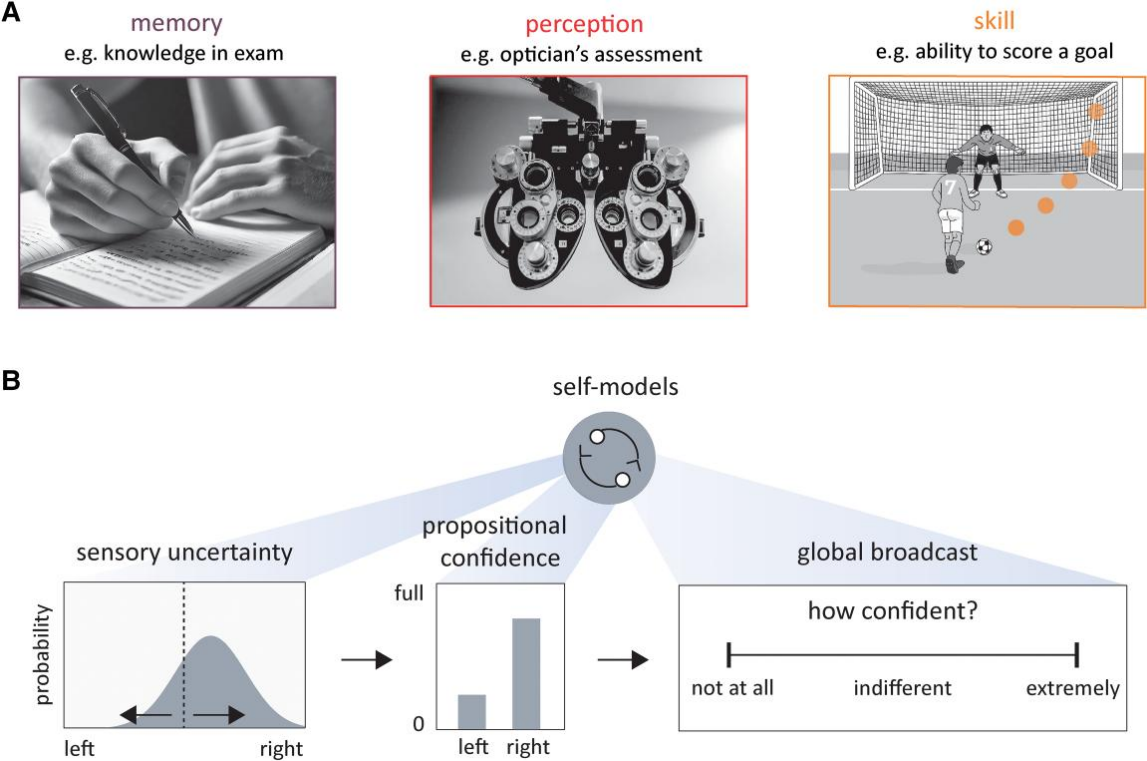
**Domains and components of metacognition.** (**A**) Real life examples of the three broad domains of metacognition: memory, perception and action or skill. (**B**) Components of metacognition include a representation of uncertainty about the qualities of a noisy stimulus. Uncertainty is then translated into a self-related frame of reference, the propositional confidence, a probability relating to a categorical decision. Confidence is then broadcast to receivers to be used for cognitive control. In experimental tasks, propositional confidence is often estimated using a visual analogue scale. Self-models of both the world and their cognitive system may or may not be accurate and influence other elements. FND could theoretically interfere with any of these components. For example, an abnormally precise prior could shift the distribution of sensory uncertainty and/or a specific defect in propositional confidence perturbs the evaluation of data in reference to self. Alternatively, in FND there may be a general shift in confidence or a non-linear amplification of confidence in either direction (akin to jumping to conclusions^14^). Abnormal ‘self-models’ could also result in abnormal high-level thoughts about oneself and the world.

Metacognition refers to cognition applied to another cognitive process (see Table 1 for dictionary of metacognitive terms).^9^ For example, when revising for an exam, it is common to evaluate what material you know and what material you don’t.^10^ If you have effective metacognition, such assessment will then provide a strategy for your future study until the confidence you have that you know all topics well enough increases. This is an example of metacognition for memory, and metacognition is equally relevant across other first-order domains such as perception or motor skill (Fig. 1A).^13^ Metacognition is the subsequent second-order judgment, a self-evaluation about how well the cognitive process unfolded or will unfold.^13^ In health, metacognitive studies give us intuitions into why and when humans change their beliefs and how humans demarcate imagination from reality.^15,16^

**Table 1:** Dictionary of metacognitive terms^9-12^

Metacognition	Cognition applied to another cognitive process
Metacognitive sensitivity	Statistical relationship between confidence judgements and objective performance
Metacognitive bias	The average confidence level
Metacognitive efficiency	A subject’s level of metacognitive sensitivity given a certain level of task performance
First-order	Task performance
Second-order	Self-evaluation and confidence in task performance
Local metacognition	Self-evaluation and confidence about a specific instance of a task
Global metacognition	Overall sense of confidence in ability

Metacognition is therefore intuitively relevant to FND as abnormal self-evaluation of cognitive processing is one mechanism via which abnormal models of self and the state of the world could arise and persist unchecked. An established neuroscientific literature maps out the different components of metacognition. Each can be experimentally examined, and each could potentially dysfunction in FND (Fig. 1B). Furthermore, if metacognition is perturbed, metacognitive retraining may help in the rehabilitation of FND. For example, in a related field of functional dyspepsia, metacognitive retraining is more effective than medication.^17^

The motivation for exploring metacognition in FND is therefore strong. Crucially, to leverage understanding using metacognitive concepts we need experiments that delineate the specific metacognitive features of functional disorders. This systematic review collates the relatively compact recent experimental literature exploring metacognition in FND. After summarizing current methods for testing metacognition, we present experimental studies armed with this prerequisite knowledge. We discuss patterns that are seen across experimental studies and whether these help us confirm or refute a metacognition-related mechanistic hypothesis. Finally, we outline unanswered questions in the hope it will stimulate fruitful research in the future.

### Estimating metacognition

**Figure 2:**
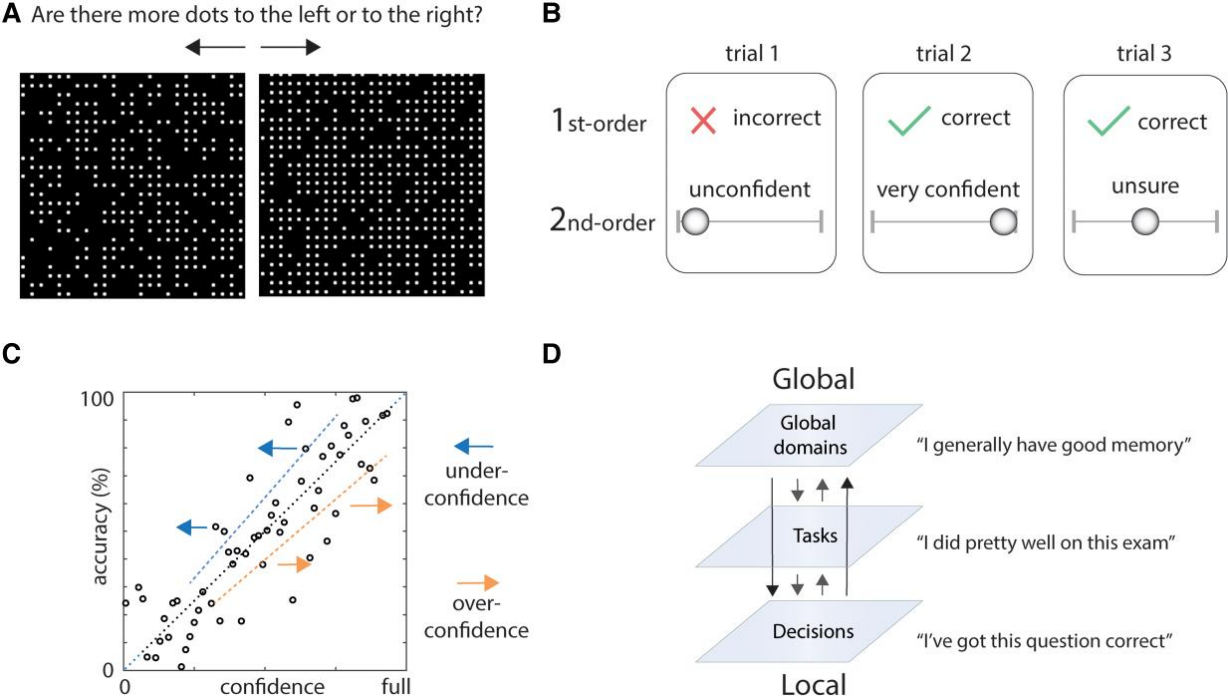
**Metacognitive experimental and analytical approaches.** (**A**) This dot task is an example of an experimental perceptual metacognitive task. Whether the black square to the left or the right has more dots allows the difficulty (and performance) to be scaled according to the difference in numbers of dots across the two squares. (**B**) After answering (either correctly or incorrectly, first-order performance), the individual then rates their confidence in their performance (second-order confidence).^10,18^ (**C**) Metacognitive sensitivity, the relationship between accuracy and confidence is plotted as a noisy correlation. Good metacognitive insight will mean that confidence increases as accuracy increases. Metacognitive sensitivity can be confounded by general bias in confidence (metacognitive bias) and performance. Under-confidence or over-confidence can lead to a general shift of individual confidence ratings, agnostic to accuracy, changing the statistical relationship. (**D**) Local confidence contributes to the formation of global self-beliefs, and global self-beliefs reciprocally influence local confidence. Each metacognitive level is associated with dynamics that unfold across different timescales, global metacognition usually has slower dynamics than local metacognition.^19^

Behavioural paradigms that test metacognition require subjects to evaluate their future or past performance on a task (example perceptual task in Fig. 2A). In most human studies, this requires subjects to assign a confidence value to a previous decision or action (‘global broadcast’ in Fig. 1B). Once there are data on a series of metacognitive judgements across a range of trials, often with graded difficulty, the statistical relationship between confidence judgements and objective performance can be examined (metacognitive sensitivity). Where metacognitive sensitivity is good, confidence should predict performance accuracy, and where metacognitive sensitivity is poor, confidence in ability does not predict performance (Fig. 2B).

As simple correlations between accuracy and confidence depend on multiple factors, a central challenge is to ensure that metrics of metacognitive sensitivity are not confounded. For example, differences in the average confidence (metacognitive bias) and differences in first-order performance influence simple correlations (Fig. 2C). A common approach is therefore to use methods and statistical measures that aim to minimize the effects of confounding influences via performance-controlled metrics. For example, in the perceptual domain, an initial experimental block can be used to scope out how sensitive an individual is to a particular stimulus. Often a staircase method is used, increasing, or decreasing the strength of the stimulus depending on the response (increase strength/decrease difficulty if wrong response, decrease strength/increase difficulty if correct response). Running subsequent experiments with stimulus strength/task difficulty set for each individual so that performance accuracy is matched across individuals and groups then allows a purer estimate of metacognition. A frequent metric used to summarize metacognition is the M-ratio or metacognitive efficiency (meta-d′/d′). Derived from signal detection theory, type 2 sensitivity (meta-d′; the ability to distinguish correct from incorrect responses) is defined using the same units as type 1 sensitivity (d′; ability to distinguish stimulus alternatives). Importantly metacognitive efficiency shows reasonable test–retest reliability across different sessions of the same experimental paradigm and across different days; and metacognitive bias has strong test–retest reliability.^10,18^ This is critical if any comparisons across groups are to be made.

Most metacognitive research in FND has focused on judgements of performance relating to specific tasks, local metacognition. A related construct is how people evaluate themselves on a broader level, global self-performance estimates, or global metacognition. Global metacognition is informed by local confidence, integrating local confidence over longer timescales to track self-performance more generally (Fig. 2D).^10-12^ Other measures have been described for examining metacognition but are yet to be tested in FND.^20^ Furthermore, continued refinement of existing metrics and models is anticipated as the assumption that the M-ratio is performance-independent has been challenged.^21,22^

Informatively, specific neural correlates of metacognitive components have been observed. For example, in both the human and animal literature, the prefrontal cortex is critical for local metacognitive confidence judgements and not task performance.^23^ In contrast, activity in the ventral striatum has been associated with subjects’ global self-beliefs irrespective of function in local confidence.^11^ Such findings hint at how the impact of experimental findings have the potential to go beyond behavioural findings and help researchers better understand the neural correlates of FND. Changes in metacognitive metrics may reveal which systems within the brain are dysfunctional in FND and which systems we should be trying to shift into a normative range with targeted interventions.

Therefore, a substantial theoretical and experimental literature suggests that metacognition is a quantifiable mechanism within the brain and can be used to help explain brain behaviour in health and understand certain disease states. Does the experimental literature point to specific metacognitive changes in FND?

## Materials and methods

We conducted a systematic review of the available literature using Preferred Reporting Items for Systematic Reviews and Meta-Analyses (PRISMA) guidelines.^24^ For full details of search terms and databases used, please refer to the Supplementary material and Supplementary Fig. 1. Broadly, the inclusion criteria were published studies that involved subjects with FND and described metacognitive findings. Review studies that commented on metacognition and FND with no new experimental data were excluded. The reference lists of relevant studies were hand searched for additional articles. After duplicates were removed, the search yielded 22 articles and 21 could be retrieved. All were evaluated for relevance after which 10 studies were included within the systematic review. Eight papers are reviewed within the manuscript, Table 2 (general measures of metacognition) and Table 3 (performance-controlled measures). All included studies were considered of sufficient quality with details of the quality appraisal given in Supplementary Table 1. As the diagnostic criteria for FND used by each included paper varied, this is detailed in Supplementary Table 2. Two related papers that did not specifically test metacognition are discussed in the Supplementary material and detailed in Supplementary Table 3. Matlab and Adobe Illustrator were used to make the figures for the paper.

**Table 2:** General measures of confidence or metacognition

Reference and participants	Tasks	Metrics	Main outcomes
Pick *et al*. (2020)^25^ Nineteen functional neurological disorder (FND): motor symptoms, sensory symptoms, dissociative seizures. Twenty healthy controls (HC). FND group significantly older. Depression and anxiety included (PHQ-9, GAD-7).	Interoceptive and exteroceptive processing task (shape counting accuracy and confidence before and after dissociation-induction procedure (sitting quietly and gazing into a large mirror for 10 min in dimly lit room)	Confidence = self-reported confidence in own interoceptive accuracy, rating from 0 (low) to 10 (high) certainty	Interoception: impairment in post-induction accuracy (*P* = 0.021, *r* = 0.379) but not baseline accuracy in FND versus HC. Lower confidence in FND versus HC at baseline (*P* = 0.001, *r* = 0.528) and post-induction (*P* < 0.001, *r* = 0.663). Exteroception: no impairment in accuracy at baseline or post-induction. Lower confidence in FND versus HC at baseline (*P* = 0.004, *r* = 0.458) and post-induction (*P* = 0.02, *r* = 0.386).
Ricciardi L. *et al*. (2021)^26^ Twenty-two functional motor disorders (FMD). Twenty-three HC. Depression and anxiety included (HADS).	Interoception task in which participants asked to count their heartbeats silently during three counting phases lasting for 25, 35 and 45 s. Recorded heartbeat captured with wrist band. Three levels of interoception assessed (accuracy, sensibility, awareness)	Cardiac interoceptive accuracy = 1/3 ∑ [1 − (recorded heartbeats − counted heartbeats/recorded heartbeats)] Interoceptive sensibility: (i) confidence self-rating between 0 = total guess and 10 = complete confidence; (ii) Body Awareness Questionnaire (BAQ) Interoceptive awareness = within participant Pearson correlation (*r*) between confidence rating and cardiac interoceptive accuracy	Lower interoceptive accuracy (*P* = 0.037, effect size 0.6) and lower interoceptive sensibility (*P* = 0.005, effect size 0.9) in functional motor disorder. No difference in metacognitive interoceptive awareness.
Bhome *et al*. (2022)^27^ Eighteen functional cognitive disorder (mean age 49.2 years, 56% male). Four hundred one HC, pre-existing validated dataset for older adults (mean age 71.4 years, 29% male).^28^ FCD group younger (*P* < 0.0001) and higher proportion male (*P* = 0.02). Co-morbid anxiety and depression (PHQ-9 and GAD-7) documented in patient group.	Global metacognition estimated using Multifactorial Memory Questionnaire (MMQ). Three sub-scales for memory ability, satisfaction and concern about memory and use of compensatory strategies	MMQ sub-score for ability and satisfaction with memory	MMQ sub-scores in FCD participants were lower (MMQ-satisfaction, *P* < −0.0001, MMQ-ability *P* < 0.0001).
Teodoro *et al*. (2023)^29^ Nineteen functional cognitive disorder (FCD). Twenty-three HC. HC had 3 more years of formal education. Significant depression excluded by using a threshold on depression questionnaire (PHQ-9) in both groups.	Stroop colour-word task (SCWT) in which ‘attentional demand’ was varied (congruent/incongruent cues within SCWT) and the presence or absence of noise (passive/active listening to oddball-type paradigm)	Subjective mental effort = sum of ‘mental demand’ and ‘mental effort’ sub-scores of a questionnaire (NASA-TLX) Objective performance = response time in modified SCWT Local metacognition = response to performance question: ‘How successful were you in accomplishing what you were asked to do?’ Self-rated after each condition. Biomarker of mental workload = suppression of EEG P300 in midline	Greater subjective effort in FCD (but not HC) when performing SCWT with noise versus silence (*P* = 0.014). Slower reaction times in FCD (mean difference 0.063 s) but no statistical task-specific effects. Accuracy equivalent. In congruent conditions patients with FCD reported worse performance in the presence of background noise (*P* = 0.016). In incongruent conditions, patients with FCD reported better performance in the presence of background noise (*P* = 0.046). EEG biomarkers were similar across tasks and groups.

Scores to estimate depression and anxiety: HADS, Health Anxiety and Depression Scale; PHQ-9, Patient Health Questionnaire-9; GAD, Generalised Anxiety Disorder-7; BAI-II, Beck Depression; and BAI, Beck Anxiety Inventories. Details of statistical comparisons: Pick *et al*., Mann-Whitney test (*P*-values) and effect size (*r*-values), Ricciardi *et al*., independent *t*-test or Mann-Whitney depending on distribution, effect size methods not stated, Bhome *et al.* independent *t*-test, no effect size, Teodoro *et al*., details not stated.

**Table 3:** Performance-controlled measures of metacognition

Reference and participants	Tasks	Metrics	Main outcomes
Begue *et al.* (2018)^30^ Ten functional motor disorders (FMD). Ten healthy controls (HC). No difference between groups in anxiety and depression (HADS).	Visuomotor (action/skill): using joystick curser to draw a straight vertical line between starting point and target. In proportion of trials, left/right deviation introduced. Patients reported: (i) if had detected the deviation; (ii) confidence about response (Likert-like scale 1 = not certain at all: 5 = very certain). fMRI examined brain activity during movement preparation and execution and confidence judgements.	Behavioural analysis: (i) accuracy of deviation detection; (ii) mean confidence in response; (iii) sensorimotor adjustment to bias (SMAB): area of deviation away from straight line (large value = lower adjustment); and (iv) average magnitude of deviation threshold Metacognitive ability: (i) over-/under-confidence score = (normalized mean confidence) − (normalized mean proportion correct); (ii) metacognitive sensitivity [area under receiver operator curve (AROC)]; and (iii) metacognitive bias	Behavioural analysis: (i) no group difference; (ii) no group difference; (iii) larger SMAB in patients = less correction than controls; and (iv) higher average threshold angle of deviation leading to correction in patients. Quantification of metacognitive ability: similar over-/under-confidence score, metacognitive sensitivity and metacognitive bias. fMRI revealed that distinct brain regions were recruited when rating confidence of their deviation detection accuracy in patients versus controls.
Verrel *et al*. (2023)^31^ Twenty-four functional movement disorders (FMD). Varied phenotypes: gait (*n* = 16), tremor (*n* = 9), tics (*n* = 5) and other (*n* = 2). Twenty-four age- and sex-matched healthy controls (HC). No patients had clinically relevant psychiatric symptoms at time of study. Depression and anxiety not assessed.	Visuomotor (action/skill) using pen on graphics tablet. One hundred ninety-two trials with: (i) centre-out movement (no visual feedback). Visuomotor deviation (randomized left/right) in blocks of 8; (ii) two candidate trajectories then shown, participant chose whether ‘actual’ or alternative trajectory. Difficulty adjusted, targeting 71% accuracy; and (iii) asked to mark confidence in decision along continuous visual analogue scale.	Movement accuracy = angular deviation from target Response accuracy = % correct trajectory judgements and signal-detection measure d′ Metacognitive sensitivity = meta-d′ Metacognitive efficiency = meta-d′/d′ (M-ratio) and Meta-AUROC (non-parametric measure of metacognitive sensitivity)	Visuomotor sensitivity (response accuracy) in the trajectory judgment was reduced in patients with FMD compared with healthy control subjects (meta-d′). No statistical difference in mean confidence and confidence range between groups. Metacognitive efficiency quantified by the M-ratio (*P* = 0.018, d_R_ = −0.76, BF_10_ = 3.09) and Meta-AUROC (*P* = 0.017, d_R_ = −0.96, BF_10_ = 3.18) significantly reduced. Subgroup analyses suggests most pronounced deficits in functional gait disturbance/functional tremor.
Matthews *et al*. (2020)^32^ Twenty functional motor disorders (FMD, 14 movement, 6 weakness), 20 matched neurological motor disorders (neuro). Twenty healthy controls (HC). Depressions and anxiety estimated (HADS).	Perceptual decision-making task used to examine: (i) attention; (ii) expectation; (iii) sensory processing (perceptual sensitivity); and (iv) metacognition.	Objective performance = type 1 signal detection theoretic measures of detection sensitivity and decision criterion Metacognitive performance = type 2 signal detection theoretic measure metacognitive sensitivity Metacognitive efficiency: meta-d′/d′ ratio (M-ratio)	Higher contrast threshold in FMD (and neuro) compared to HC. Detection sensitivity and decision criterion equivalent for FMD and HC (but differs in neuro). Metacognition is equivalent between groups (both metacognitive sensitivity and efficiency).
Bhome *et al*. (2022)^27^ Eighteen functional cognitive disorder (FCD, *n* = 14 in this task). Web group: 54 HC from pre-existing web study. Age but not sex matched and task different. NYU group: 30 HC from a neuroimaging study, significantly younger.	Local metacognition tested for memory (working) and perception (vision). Forced choice response with staircase to keep task accuracy at 70–72% followed by a confidence rating.	Metacognitive efficiency = meta-d′/d′ ratio (M-ratio)	Local metacognitive efficiency equivalent to controls for both memory and perception task.
Pennington *et al*. (2021)^33^ Twenty FCD. Fourteen neurodegenerative mild cognitive impairment (nMCI). Twenty-three HC. Mild low-mood and anxiety included.	Forced choice memory (words on screen) and perceptual task (estimating circle containing most dots) with trial-by-trial confidence ratings (1.00 = low to 6.00 = high).	Metacognitive efficiency: meta-d′/d′ ration (M-ratio)	No metacognitive differences between groups, either on the memory or perceptual task.

Abbreviations common to Table 2. Details of statistical comparisons: Begue *et al*., ANOVA (2 × 2 × 2) to assess main effects of group, deviation of trajectory and detection. Group comparison for over-/under-confidence not clear. Verrel *et al*., Mann–Whitney, effect size robust Cohen’s (d_R_) and Bayes Factor (BF_10_), Matthews *et al*., Bayesian mixed ANOVA, Bhome *et al.* hierarchical Bayesian modelling, 95% high density interval from sampled estimates of posteriors, Pennington *et al*., linear regression analysis for between-group analysis controlling for age and sex.

## Results

### Metacognition: general measures

In this section, we detail the papers that have studied the relationship between accuracy and confidence (not controlling for performance across groups) or estimated metacognition using other methods such as questionnaires (Table 2).

Pick *et al.*^25^ tested confidence in two tasks, a mixed group of patients with FND and healthy controls before and after dissociation induction (using a mirror-gazing technique). An interoception task involved counting heartbeats, and self-reported confidence in their interoceptive accuracy was scored between 0 (low) and 10 (high) certainty. A computerized geometric shape counting task was also completed, and their confidence in their shape counting accuracy was scored similarly. All measures of confidence were significantly lower in the patient group compared to controls (pre/post dissociation induction, across tasks). The accuracy in the interception task was lower in the patients compared to controls only after dissociation induction, suggesting a decoupling of confidence and performance (confidence low across conditions, performance only low after dissociation), but performance-controlled metrics were not computed. Overall, the study suggested that ‘measures of confidence’ in perceptual and cognitive tasks were low in this mixed group of patients with FND.

Another study examining interoception was conducted by Ricciardi *et al*. Both cardiac interoceptive accuracy and patients’ confidence in their perceived accuracy (‘interoceptive sensibility’) were lower in functional ‘motor’ disorders.^26^ However, they found no difference across groups in the relationship between accuracy and confidence, using a correlation to estimate ‘metacognitive sensitivity’ (‘interoceptive awareness’, the measure of metacognition in this analysis).

Bhome *et al.*^27^ estimated ‘global’ metacognition by asking participants to complete the Multifactorial Memory Questionnaire (MMQ, performance-controlled measures from this paper discussed in next section). MMQ-ability measures self-perception of everyday memory ability and MMQ-satisfaction measures overall appraisal of one’s own memory. Both mean MMQ-ability and mean MMQ-satisfaction were lower in functional ‘cognitive’ disorder, and this was interpreted as indicating worse global metacognition. Of note, demographic differences across groups could have confounded results. For example, the patient group were much younger (mean age 49.2 years) than the normative comparison group (mean age 71.4 years) and unusually there were a higher proportion of males in the patient group compared to the control group. Higher depression scores in functional ‘cognitive’ disorder were significantly associated with lower MMQ sub-scores.

Teodoro *et al.*^29^ tested subjective mental effort, attentional reserve and performance in patients with functional cognitive disorder and healthy controls. The experiment involved a Stroop Colour-Word Task (SCWT) in which attentional demand was varied using congruent and incongruent cues within the SCWT and the presence or absence of noise (passive/active listening to an oddball-type paradigm). The ‘metacognition’ metric was the score on the item ‘Performance’ on the NASA-TLX question ‘How successful were you in accomplishing what you were asked to do?’; the scale requires participants to rate their performance on a visual analogue scale with ‘Perfect’ on the left and ‘Failure’ on the right. This is a potentially counterintuitive assignment to the ‘left’ and ‘right’ sides that could be misinterpreted. Participants with functional cognitive disorders reported ‘worse’ performance on the questionnaire in congruent SCWT conditions in the presence of background noise. For incongruent conditions, participants reported ‘better’ performance in the presence of background noise. These patterns contrasted the behavioural patterns seen in healthy controls.

### Metacognition: performance-controlled

This section summarizes the five papers that estimated metacognition with performance-controlled methods relating to specific tasks (local metacognition, Table 3). This allows first-order performance and metacognitive function to be estimated independently yielding more certainty about where deficits reside.

**Figure 3:**
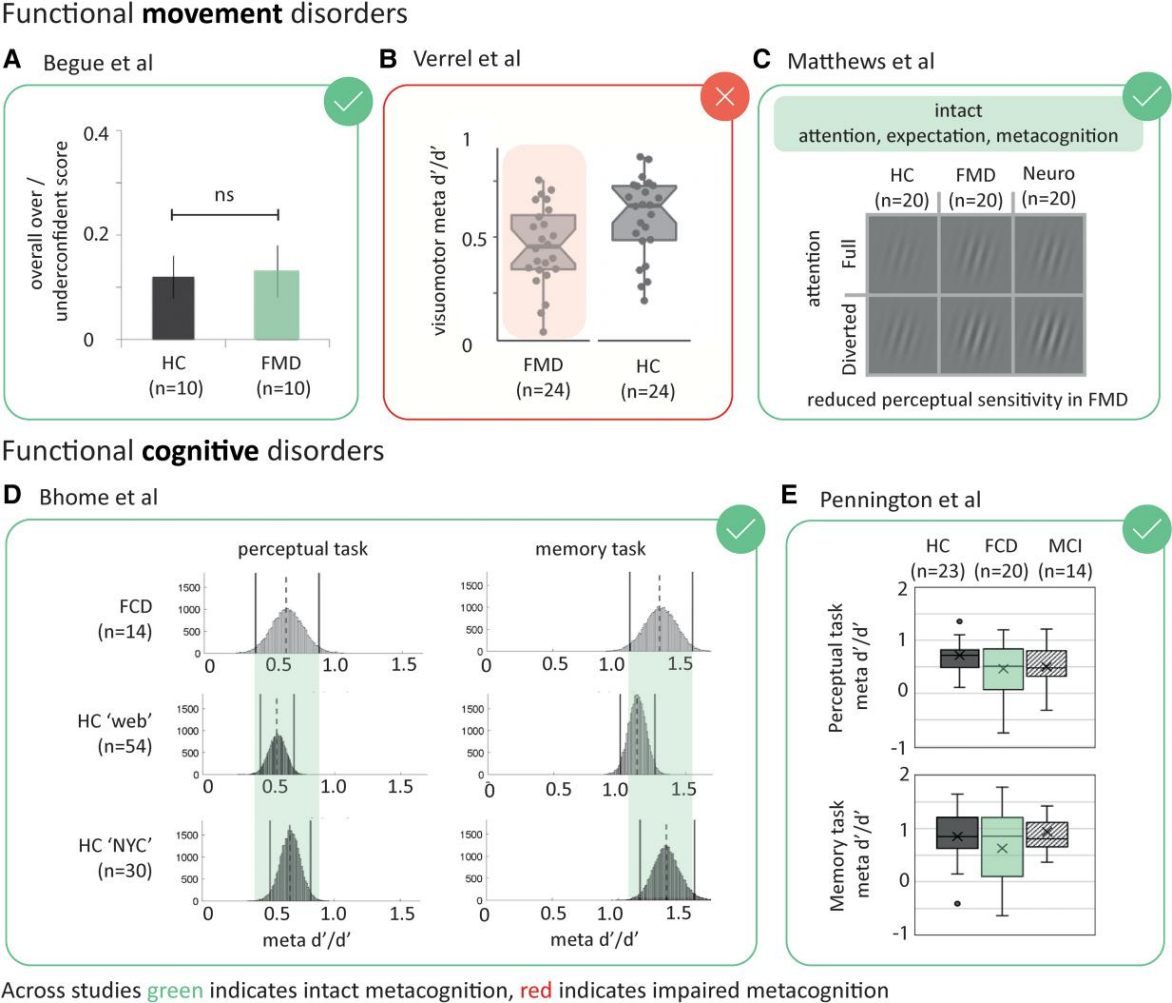
**Graphical summary of performance-controlled studies of metacognition in functional neurological disorders (FNDs).** Across studies, green ticks indicate intact metacognition, red crosses impaired metacognition. Full details of study participants, tasks, metrics and main outcomes are given in Table 3. The numbers of each group are given in brackets below labels (*n*). (**A**) Begue *et al*. In a visually guided motor task, first-order performance was impaired in patients with mixed functional movement disorders (FMD) compared to healthy controls (HC). However, their confidence scaled appropriately with accuracy: average confidence ratio and measures of metacognitive sensitivity were intact. The plot shows overall over-/under-confidence score is plotted as a bar plot with error bars representing standard error of the mean for each group [not significant (ns)].^30^ (**B**) Verrel *et al*. Patients with FMD had lower visuomotor sensitivity (first-order performance) and reduced metacognitive performance (meta-d′/d′) compared to healthy controls in a sensorimotor task. The group result is shown as a boxplot, individual datapoints plotted as a dot, and the group comparison was statistically significant (Mann–Whitney *P* = 0.018, effect size robust Cohen’s = 0.76). (**C**) Matthews *et al*. In a visual perceptual task, attentional, expectational and metacognitive mechanisms were intact in FMD (Bayesian mixed ANOVA), but they required higher visual contrast than controls to maintain the same detection sensitivity, suggesting that first-order performance was impaired. Magnitude of first order impairment is shown by the differences in the mean Gabor contrast required to achieve 79.4% detection sensitivity under full and diverted attention for each group.^32^ (**D**) Bhome *et al*. In a memory recall task and a perceptual task, patients with functional cognitive disorders (FCDs) demonstrated intact metacognitive efficiency (meta-d′/d′) comparative to two healthy control groups (hierarchical Bayesian modelling).^27^ Each plot shows sample count on the *y*-axis versus meta-d′/d′ on the *x*-axis, and groups were compared using. (**E**) Pennington *et al*. In a memory task and visuospatial perception task, task performance differed in patients with functional cognitive disorder (FCD) and neurodegenerative mild cognitive impairment (MCI) compared to HC, but confidence ratings suggested no difference in local metacognition between groups. Box plots show meta-d/d for each group in perceptual and memory tasks (linear regression analysis for between-group analysis controlling for age and sex).^33^

Begue *et al.*^30^ was the first to examine metacognition in FND. Taking a small group of patients with mixed functional ‘movement’ disorder and age-matched controls, participants moved a joystick to draw a straight line on a screen. In a proportion of trials, a right/left deviation was introduced during the movement and participants were asked to adjust their movement so that they continued to draw a straight line, compensating for the deviation. As there were insufficient task repetitions to allow the calculation of an M-ratio, the assessment of metacognition relied on alterative measures [such as the over-/under-confidence in performance score (Table 1)]. Begue *et al.* found that first-order performance was impaired in patients, with reduced adjustments to deviations and a greater angle of deviation required for detection. However, their ‘average confidence’ ratio and measures of ‘metacognitive sensitivity’ were intact; their data suggested that confidence scaled appropriately with accuracy (Fig. 3A).

Contrasting results were found by, Verrel *et al.*^31^ in a study of patients with a range of functional ‘movement’ phenotypes and an equal number of healthy controls. Again, a ‘visuomotor’ task was used that required centre-out movements of a pen on a graphics tablet, and a visuomotor deviation was used to introduce error. After every trial, participants were required to identify their preceding movement out of a choice of their actual movement and a distorted alternative. They then rated their confidence in their movement identification judgement. Verrel *et al.* found that patients with functional ‘movement’ disorders had lower visuomotor sensitivity (first-order performance) and reduced metacognitive performance compared to healthy controls. ‘Reduced metacognitive efficiency’ was confirmed via two independent analyses (M-ratio with a medium effect size of 0.76 estimated using robust Cohen D (Fig. 3B) and ‘Meta-AUROC’ with large effect of 0.96). Care was taken over differences in baseline performance between groups (such as increased response variability), so that as far as possible, such effects were either controlled for or acknowledged. Reduced metacognitive efficiency was particularly interesting as general ratings of confidence (both mean and variation) were equivalent across groups suggesting a specific metacognitive effect. In summary, this study appeared to effectively use metacognitive paradigms and analysis, with a task design (sensorimotor) that mapped to the clinical phenotype of a functional motor deficit and found both first-order and metacognitive performance to be different to controls.

Matthews *et al.*^32^ extended these findings in functional ‘movement’ disorders with a visual perceptual task in which they ambitiously examined four core cognitive domains implicated in FND: attention, expectation, sensory processing (perceptual sensitivity) and metacognition. They carefully adjusted the sensory inputs to provide a performance-controlled stimulus contrast for each individual. Interestingly, patients with functional movement disorders exhibited statistically equivalent attentional, expectation and metacognitive processing to healthy controls. However, first-order performance was impaired and patients with functional ‘movement’ disorders, and patients with phenomenologically matched neurological motor disorders, required higher visual contrast than controls to maintain the same detection sensitivity (relative stimuli contrast differences shown in Fig. 3C). Matthews *et al.*, therefore, concluded that ‘higher’ attentional, expectational and metacognitive mechanisms are intact in FND whereas basic sensory processing may be impaired.

In addition to global metacognitive estimates, Bhome *et al.*^27^ also tested local metacognitive efficiency in patients with functional ‘cognitive’ disorder. Healthy controls were comprised of two groups: an age-matched ‘web’ group taken from a larger pre-existing study with some differences in the psychophysical task tested, and a New York University (NYU) group that were not age matched from a previous neuroimaging study that had completed a similar psychophysical task (mean control age = 24.9 years, mean patient age = 49.2 years). To test ‘local’ metacognition, subjects were presented with an array of stimuli for 2 s to memorize and then asked to recall whether an item was present using a forced choice response. A staircase procedure kept the task accuracy equivalent across group. Each decision was followed by a confidence rating (visual analogue scale of ‘complete guess’ through to ‘absolutely certain’). A similar design across two scenarios allowed metacognitive efficiency for both a memory and perception task to be estimated. This study found that ‘metacognitive efficiency’ was intact in the patient group. However, differences in control demographics and testing (‘web’ group task differences, ‘NYU’ group half the age of patient group) call into question the validity of the control group (Fig. 3D).

Finally, Pennington *et al.*^33^ also used a memory task and a visuospatial perception task with patients with functional ‘cognitive’ disorder and compared their metacognition to patients with neurodegenerative mild cognitive impairment and healthy controls. Previous studies have shown that once patients have a diagnosis of ‘dementia’, poor insight into their memory loss is paralleled by a loss of metacognitive awareness within experimental paradigms; this study extended these finding by examining the milder neurodegenerative subtype.^34^ A forced choice memory task involved the participants recalling words they had been shown on screen and distinguishing these from distractor words. The perceptual task involved viewing circles containing dots and estimating that had the greater number of dots. These were accompanied by trial-by-trial confidence ratings, ranging from ‘1’ as the lowest rating confidence through to ‘6’ as the highest rating of confidence. Again, in this study, no metacognitive differences emerged between the groups. This was in the context of weaker performance by the functional cognitive disorder and neurodegenerative mild cognitive impairment groups on the memory task. This result suggested that the primary difficulty experienced by both groups was in completing the task (first-order) rather than evaluating their performance (second-order, Fig. 3E).

### Summary of literature

In summary, five studies used performance-controlled metrics, the gold-standard methods in neuroscientific research, to estimate local metacognition in FND. The required design and analysis are more complicated but this investment in data allows confounders such as differences in performance and potential cognitive biases to be controlled and/or modelled with metrics such as metacognitive efficiency (M-ratio). In functional movement disorders, two studies tested visuomotor paradigms with results for and against intact metacognition; a single study in the perceptual domain found intact metacognition. The two studies in patients with functional cognitive disorder have shown intact metacognitive efficiency for memory and perceptual memory tasks. Thus, collectively, current experimental evidence supports intact metacognition in the cognitive domain and there is equivocal evidence in the motor domain. Metacognitive sensitivity (not performance-controlled) for interoceptive judgements has also been examined and appears intact in functional movement disorders. Although we did not set out to review general confidence in FND, a mixed picture of normal or low confidence has been documented within the literature evaluated in this systematic review.

## Discussion

Metacognition, a measure of how accurately our self-evaluation maps onto the reality of our performance across cognition, perception and action, is instinctively relevant to FND. In FND, abnormal beliefs about the state of the brain, body and world are thought to seed throughout the brain blocking healthy performance. If self-evaluation could shine a light on dysfunctional processing in FND, metacognition is a potential ‘way in’ to flag erroneous processes. We also sorely need experimental tools to quantify and understand core axes of mechanism in FND, particularly if quantification can be reached using non-invasive, inexpensive computer-based metacognitive tasks. The enthusiasm that metacognition may give substance to FND’s intangible nature is therefore well justified.

However, overall, this systematic review reveals that most papers find metacognition is equivalent to healthy controls, and one paper supports the notion that metacognition is impaired. We therefore try and broaden the debate to discuss alternative potential scenarios that link metacognition and FND.

If metacognition is impaired in FND, the single ‘positive’ study may give us some clues for how. For example, the task was a visuo‘motor’ metacognitive paradigm that mapped to the phenotype of a functional ‘movement’ disorder, hinting that the domain of symptoms in FND may predict the nature of the local metacognitive deficit. Another more speculative observation is that the confidence judgement was the ability to recognize ones’ own performance rather than an evaluation of performance itself. Therefore, the metacognitive decision could be considered a ‘third-order’ metacognitive judgement or meta-metacognition.^35^ Humans are considered to have the capacity to perform repeated hierarchical evaluations of their performance up to at least fourth-order judgements (i.e. meta-meta-metacognition) and such segregations may be relevant in FND.^36^

There are also many potential reasons why the data do not yet support intuitions that metacognition is important in FND (false negative or type II error). For example, several studies try to control for co-morbidities such as anxiety, depression and pain, on the assumption that we should sample experimental correlates for FND rather than the correlates of the mixed clinical picture. Some studies selected patients ‘without’ any documented co-morbidities, an approach that is poorly representative of FND as co-morbidities in FND are common. Controlling for the influence of co-morbidities is also problematic as co-morbidities may innately interact and sculpt the disorder at all levels of mechanism. Furthermore, anxiety and depression are linked to lowered confidence and the converse with compulsivity.^19^ Comparing FND to other patient groups with similar levels of co-morbidities may be informative in the future. A related topic is whether performance-controlled metrics should have gold-standard status in FND. This critically depends on how FND corrupts information processing more generally within the brain. Studies in this systematic review^31-33^ and others^37^ suggest impaired first-order processing in FND. How to control for this, and ensure that metacognition is independently modelled, is complicated. Including variation in task difficulty, across conditions and/or across groups, runs the risk of inflating or conflating metacognition in one group relative to the other. Lastly, it is well recognized that FND represents a very heterogeneous group. Furthermore, different diagnostic criteria for FND were described amongst the included studies that may have increased variability within the findings. It may be that selective impairments in some are hidden within the population variability. For example, autism spectrum disorder is both over-represented in FND and associated with metacognitive dysfunction suggesting a potential interplay.^38^ We need further studies to better delineate if a specific metacognitive profile is universally relevant for FND or more relevant for particular cohorts with specific aetiological features and/or risk factors.

An alternative headline that is infrequently debated is whether metacognition is intact given all the null studies. One fascinating scenario builds from the evidence presented in this systematic review of impaired first-order and intact second-order metacognition. For example, in the perceptual domain studies have shown that if patients with FND are tested using an equally salient sensory experience (performance-controlled), then patients’ metacognitive judgments are equivalent to controls. The first-order processing, incoming data informing metacognition may be abnormal in FND. However, if performance-controlled metrics are used this still allows the independent evaluation of intact metacognition. As such explicit metacognition paradigms quantify how patients correctly scale their confidence based on the information that is consciously available, intact metacognition potentially offers a quantification of the fact that patients are not ‘putting anything on’. If confirmed in further studies, intact metacognition in FND would contrast with other core cognitive and psychiatric disorders that can be differentials for FND in which metacognitive deficits are seen.^39^

One thing that is certain is that the relationship between metacognition and FND does not appear to be a simple one and underestimating the complexity of the topic will likely produce noisy results in the future. To date, questionnaire metrics have been used to estimate elements of global metacognition and a considerable narrative built from very mixed data. The unidimensional output from questionnaires is often a value in response to a single question or averaged sub-score over a sequence of questions. Such values poorly capture multidimensional constructs such as self-belief on a topic. Control group selection is also important as demographic factors influence metacognition (e.g. age^40,41^ and sociocultural influences^42^). Subgroups of FND will also be more vulnerable to confounding factors, owing to its multifactorial aetiology. Future studies can also aim to have translational impact so that metacognitive science is not only descriptive. Ongoing therapeutic trials of metacognitive training combined with neuro-physiotherapy will no doubt reveal new information.^43^ This review has revealed open questions about what the specific pattern of metacognitive change is in FND. Thus, although we are not yet at a point where we can use metacognitive retraining as a targeted intervention to shift a characteristic ‘fingerprint’ of FND metacognitive dysfunction, metacognitive retraining may be a helpful general mechanism for greater cognitive self-awareness that interacts positively with axes of mechanism in FND.

Finally, until we better appreciate whether FND is associated with specific metacognitive profiles, it may be premature to try and reliably map metacognitive science to other FND aetiological models the implicate core constructs such as attention, self-agency, emotion/threat processing and hierarchical predictive coding. Some specific studies may help this discussion, for example, a recent study suggested that judgements of agency do not recruit metacognitive processing, putting a distance between a core FND feature and metacognition.^44^ However, other studies in healthy controls suggest potential links; a recent study that quantified expectations (priors) and metacognition found that when higher expectations for strong (perceptual) signals are present, individuals are more confident with no change in objective performance.^45^ Metacognition and FND are both hard topics to study and the ideas and phenomena that one is attempting to quantify can be nebulous. For this reason, we have tried to highlight metacognitive work that has used robust experimental tools that have good evidence behind their use through many years of development in human and animal work. Making sure that a good dialogue exists between neuroscientists that lead metacognition research and clinical researchers that have an equal grip on the full scope of FND will likely accelerate understanding and encourage cross talk between aetiological frameworks that offer alternative perspectives on FND.

In summary, the momentum behind metacognitive research is obvious. This systematic review has tried to provide an analytical checkpoint after the first five years of experimental work, an evidence architecture for where we currently are and flag the many unanswered questions that future work will help us address.

## Supplementary Material

fcaf014_Supplementary_Data

## Data Availability

Please see Supplementary material for the detailed search strategy and overview of related experimental studies. Data sharing is not applicable to this article as no new data were created or analysed in this study.
