# Supplementary material for: Going ‘meta’: a systematic review of metacognition and functional neurological disorder
Source: Brain Commun. 2025 Jan 13;7(1):fcaf014. doi: 10.1093/braincomms/fcaf014 (PMC11775622; doi:10.1093/braincomms/fcaf014)
Supplement: fcaf014_Supplementary_Data [file fcaf014_supplementary_data.docx]

**Supplementary material**

**Search strategy and results**

A search was conducted using the following databases: Embase (1974 to 2023 Week 16), Ovid MEDLINE(R) ALL (1946 to April 21, 2023), and APA PsycInfo (1967 to April Week 3 2023). Given the range of terms used to describe functional neurological disorder and its diverse phenotypes, the search strategy included the exploded MeSH terms: functional neurological disorder, functional motor disorder, functional movement disorder, functional cognitive disorder, functional weakness, functional tremor, functional dystonia, functional gait, functional sensory, functional speech, functional swallow, functional tic, functional parkinsonism, functional myoclonus, functional seizures, conversion disorder, psychogenic, non-epileptic, dissociative neurological, dissociative neurological symptom disorder, and dissociative seizures. These terms were combined with exploded MeSH terms: metacognition, metacognitive, self-cognizance, self-cognizant, meta-perception, and meta-memory. The search was limited to English language articles and adult subjects.

The inclusion criteria were: published studies with data collection that involved subjects with FND and described metacognitive findings. The reference lists of relevant studies were hand searched. After duplicates were removed, the search provided 22 articles. One article could not be accessed.^1^ Using our inclusion criteria, four articles were excluded due to low relevance; these were a paediatric case study,^2^ a survey of healthy adults,^3^ a study of acquired brain injury^4^ and a review of interventions for dissociative disorders.^5^ Review studies that commented on metacognition and FND with no new experimental data were not included.^6-12^

**Supplementary Figure 1. Details of search strategy**


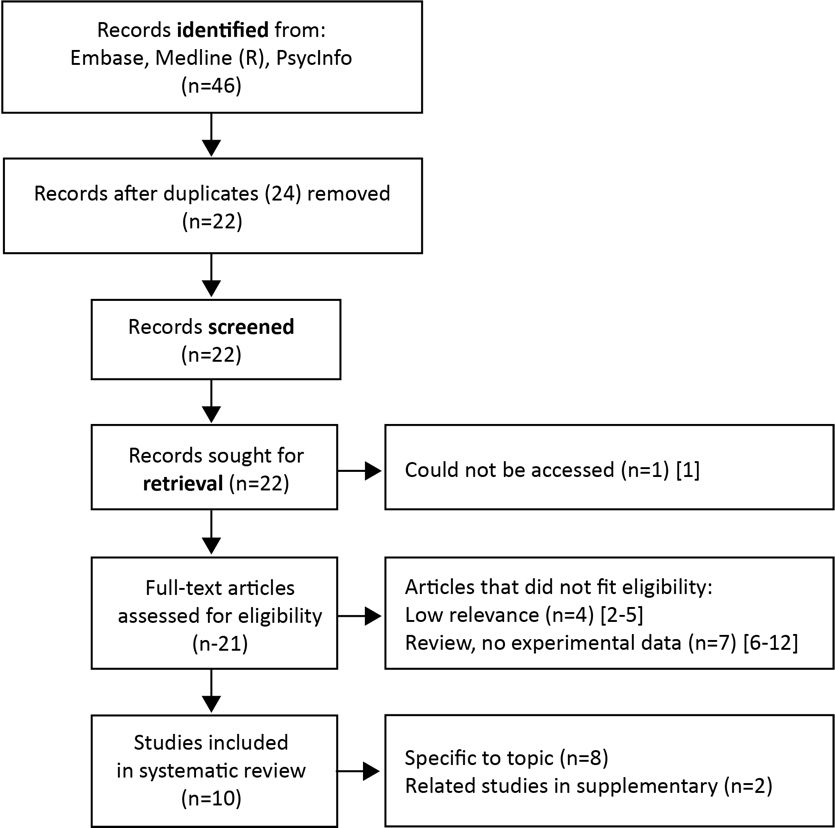


**Quality appraisal**

10 articles were included in systematic review. A checklist comprising nine questions was devised to help appraise the quality of each article. The questions were adapted from a checklist for cohort studies recommended by the Critical Appraisal Skills Programme (CASP)^13^, and a systematic review appraisal sheet developed by the Centre for Evidenced-Based Medicine^14^. Each paper was assessed nine criteria on a checklist **(Supplementary Table 1).**The nine criteria were: 1. A clear research question, 2. Appropriate recruitment, 3. Valid methods, 4. Valid measurements, 5. Identification of confounding factors, 6. Adequate consideration of confounding factors in design/ analysis, 7. Confidence in the results, 8. Validity of results, and 9. Relevance to the patient population. Three of the authors met to discuss each article in detail, to ensure the appraisal was balanced. The 0 - 1 binary scale required a criteria to be demonstrated fully to be awarded a score of 1 (no partial scoring) and there was a maximal score of nine. The ten articles reviewed were all deemed sufficient quality to include. In general, articles demonstrated a clear research focus, awareness of known confounding factors, and were relevant to the FND population. Lower scoring articles either did not demonstrate sufficiently their rationale for the chosen measurement to address the research question or minimise bias, or their rationale for the recruitment method, or task design, to adequately address confounding factors that may have influenced results.

**Details of diagnostic criteria used**

The differences in diagnostic criteria for FND used across studies are summarised in **Supplementary Table 2.**

**Metacognition: related findings**

Two related papers were also identified and both had null findings **(Supplementary Table 3).** **Jungilligens *et al.****,* looked at emotional, behavioural and interoceptive awareness in patients with dissociative seizures and healthy controls.^15^ The authors focused on the awareness of movement in a Libet clock paradigm (rather than confidence judgements) and found behavioural awareness to be intact. **Huys *et al.*,** compared subjective to objective tremulousness (perceived symptoms vs actual symptoms) in participants with a functional action tremor, patients with neurological action tremor and a healthy control group.^16^ In the task, participants moved their index finger on a touchpad from a starting position to a target straight ahead and this was viewed on a screen. Subjects had direct vision of the touchpad and their arm in the 'real-time' task. In the 'retrospective' task, their arm was hidden underneath a cover. During the task, lateral deviation of the near-average trajectory was distorted, and participants chose the trajectory they thought corresponded most closely to their average of all previous trials. A gain of one indicated no distortion. ‘Retrospectively’, all groups overestimated the tremor/non-straightness of their reaching trajectories. A statistical comparison of the difference of subjective compared to objective tremor severity across the three groups was not significant. In ‘real-time’ conditions healthy controls were found to underestimate the amount of tremor (perceived gain = 0.69) and neurological tremor patients were also found to underestimate but to a lesser degree (perceived gain = 0.82). Interestingly, those with a functional tremor had a near perfect gain with no distortion for actual versus perceived tremor (perceived gain = 1.04). Statistically this effect was not validated once depression and anxiety were controlled for, but as we will discuss, whether we should be controlling for anxiety and depression is a complex topic. In contrast to studies exploring metacognitive sensitivity, participants were not asked to judge their confidence of their ‘real-time’ perceptions of tremulousness. However, within a broad definition of metacognition, this study hints that there may be an altered perceptual judgement of movement.

**References**

1. Inanir S, Taycan SE, Inanir A, Celikel FC, Etikan I. Metacognitive evaluation in patients with fibromyalgia syndrome. *Klinik Psikofarmakoloji Bulteni* 2015; **25**: pS175-S. 2/3p.

2. Robinson S, Hedderly T. Novel Psychological Formulation and Treatment of "Tic Attacks" in Tourette Syndrome. *Front Pediatr* 2016; **4**: 46.

3. McWhirter L, King L, McClure E, Ritchie C, Stone J, Carson A. The frequency and framing of cognitive lapses in healthy adults. *CNS Spectr* 2022; **27**(3): 331-8.

4. Rotenberg-Shpigelman S, Rosen-Shilo L, Maeir A. Online awareness of functional tasks following ABI: the effect of task experience and associations with underlying mechanisms. *NeuroRehabilitation* 2014; **35**(1): 47-56.

5. Subramanyam AA, Somaiya M, Shankar S, et al. Psychological Interventions for Dissociative disorders. *Indian J Psychiatry* 2020; **62**(Suppl 2): S280-S9.

6. McWhirter L, Ritchie C, Stone J, Carson A. Functional cognitive disorders: a systematic review. *Lancet Psychiatry* 2020; **7**(2): 191-207.

7. McWhirter L, Carson A. Functional cognitive disorders: clinical presentations and treatment approaches. *Pract Neurol* 2023; **23**(2): 104-10.

8. Cretton A, Brown RJ, LaFrance WC, Jr., Aybek S. What Does Neuroscience Tell Us About the Conversion Model of Functional Neurological Disorders? *J Neuropsychiatry Clin Neurosci* 2020; **32**(1): 24-32.

9. Bhome R, McWilliams A, Huntley JD, Fleming SM, Howard RJ. Metacognition in functional cognitive disorder- a potential mechanism and treatment target. *Cogn Neuropsychiatry* 2019; **24**(5): 311-21.

10. Larner AJ. Functional Cognitive Disorders (FCD): How Is Metacognition Involved? *Brain Sci* 2021; **11**(8).

11. Bègue I. From emotion processing to metacognition. *Swiss Archives of Neurology* 2018; **169**: 253-9.

12. Larner AJ. Dementia screening: a different proposal. . *Future Neurology* 2018; **14**: 177-9.

13. Critical Appraisal Skills Programme. 2023. <https://casp-uk.net/casp-tools-checklists/>.

14. Centre for Evidenced-Based Medicine. 2023. <https://www.cebm.ox.ac.uk/resources/ebm-tools/critical-appraisal-tools>

15. Jungilligens J, Wellmer J, Schlegel U, Kessler H, Axmacher N, Popkirov S. Impaired emotional and behavioural awareness and control in patients with dissociative seizures. *Psychol Med* 2020; **50**(16): 2731-9.

16. Huys AML, Haggard P, Bhatia KP, Edwards MJ. No exaggerated tremor severity perception in functional tremor. *J Neurol* 2022; **269**(11): 6043-8.

17. Fahn S, Williams DT. Psychogenic dystonia. *Adv Neurol* 1988; **50**: 431-55.

18. Ball HA, McWhirter L, Ballard C, et al. Functional cognitive disorder: dementia's blind spot. *Brain* 2020; **143**(10): 2895-903.

19. Diagnostic and statistical manual of mental disorders: DSM-5-TR. In: Association AP, editor.; 2022.

20. Gasca-Salas C, Lang AE. Neurologic diagnostic criteria for functional neurologic disorders. *Handb Clin Neurol* 2016; **139**: 193-212.

**Supplementary Table 1. Quality Assessment of studies included in systematic review.**

| **Quality Appraisal Checklist for literature on metacognition and FND** | |  |  |  |  |  |  |  |  |  |  |
| --- | --- | --- | --- | --- | --- | --- | --- | --- | --- | --- | --- |
| **Scoring: Yes = 1, No/unable to determine = 0** | |  |  |  |  |  |  |  |  |  |  |
|  | **First author/ published** | **Pick (2020)** | **Ricciardi (2021)** | **Bhome (2022)** | **Teodoro (2023)** | **Begue (2018)** | **Verrel (2023)** | **Matthews (2020)** | **Pennington (2021)** | **Jungilligens (2020)** | **Huys (2022)** |
| 1 | Did the study address a clearly focused issue or question? (population studied and outcomes considered) | 1 | 1 | 1 | 1 | 1 | 1 | 1 | 1 | 0 | 1 |
| 2 | Was the cohort recruited in an acceptable way? (representative of a defined population, any risk of selection bias) | 1 | 1 | 0 | 0 | 1 | 0 | 1 | 1 | 1 | 1 |
| 3 | Did the study use valid methods to address the question? | 0 | 0 | 1 | 1 | 1 | 1 | 1 | 0 | 0 | 0 |
| 4 | Was the outcome accurately measured to minimise bias? (were subjective or objective measurements used, did the measurements reflect what they were meant to) | 0 | 1 | 0 | 1 | 0 | 1 | 1 | 0 | 1 | 1 |
| 5 | Have the authors identified all important confounding factors? (did the authors miss any) | 1 | 1 | 1 | 1 | 1 | 1 | 1 | 1 | 1 | 1 |
| 6 | Have the authors taken account of confounding factors in the design and/or analysis? (e.g. modelling, stratified-, regression-, or sensitivity analysis to correct, control or adjust for confounding factors) | 1 | 0 | 1 | 1 | 0 | 1 | 1 | 0 | 1 | 0 |
| 7 | Are the results precise? (the rate difference, range of confidence intervals) | 1 | 1 | 1 | 1 | 1 | 1 | 1 | 0 | 0 | 1 |
| 8 | Do you believe the results? (a big effect is hard to ignore, can it be due to chance or confounding, is there bias due to selection of results or missing data) | 1 | 1 | 1 | 1 | 0 | 1 | 0 | 1 | 1 | 0 |
| 9 | Can the results be applied to our patient population? (were the subjects sufficiently similar) | 0 | 1 | 1 | 1 | 1 | 1 | 1 | 1 | 0 | 1 |
|  | **Total (max 9)** | **6** | **7** | **7** | **8** | **6** | **8** | **8** | **5** | **5** | **6** |

**Supplementary Table 2. FND Diagnostic criteria used across included studies.**

| **Study** | **FND diagnostic criteria used** |
| --- | --- |
| Pick et al 2020 | Did not state which diagnostic criteria were used |
| Ricciardi et al 2021 | Clinically Established and Documented, according to Fahn and Williams criteria^17^ |
| Bhome et al 2022 | Functional Cognitive Disorder Diagnostic Criteria described by Ball et al^18^. |
| Teodoro et al 2023 | Did not state which diagnostic criteria were used |
| Begue et al 2018 | DSM 5 criteria^19^ |
| Verrel et al 2023 | Based on positive clinical signs as described by Gasca-Salas et al^20^ |
| Matthews et al 2020 | DSM 5 criteria^19^ |
| Pennington et al 2021 | Based on clinical presentations with discrepancy between symptoms and observed or reported everyday cognitive functioning. No specific criteria were stated. |

**Supplementary Table 3. Related findings in FND**

| **Reference and Participants** | **Tasks** and links to questionnaires | **Metrics and** links to data/code | **Main outcomes** |
| --- | --- | --- | --- |
| **Jungilligens et al. (2020)^15^**  20 with *dissociative seizures* (DS)  20 HC  *DS patients had more clinically relevant depression and anxiety than HC (BDI-II, BAI)* | **Emotional recognition**, awareness and regulation: custom-made go/no-go task that cues depending on recognition of emotional vs neutral faces. **Behavioural awareness**: digital Libet clock: on rotating clock indicate either will to move (W-judgement - 40 trials) or when key pressed (M-judgement – 40 trials).  **Interoceptive awareness:** heartbeat counting paradigm. |  | **Emotion recognition** impaired in DS vs HC.  **Behavioural inhibition** reduced in DS vs HC.  **Behavioural awareness**: whole task within normal limits.  Intact **interoceptive sensitivity** |
| **Huys et al. (2022)^16^**  21 with functional *tremor*  21 with organic tremor  23 HC  *Depression and anxiety assessed across groups (HADS)* | Compared subjective to objective tremulousness. Aimed to use a task that excluded potential confounding metacognitive factors (such as circumstances, expectations, mood). | Retrospective tremor judgement (indirect visual feedback)  Retrospective tremor judgement (direct visual feedback)  Real-time tremor perception  . | Retrospectively, all three groups overestimated their tremulousness. No significant difference between the three groups.  Real-time tremor perception was significantly different across groups (healthy controls > organic tremor underestimated tremor). Statistical effect collapsed when higher anxiety and depression in patient group were co-factors in statistical model. |

These studies use paradigms that are not commonly considered under the metacognitive rubric but may inform discussion.
